# Evaluation of cellular changes in blood stored for transfusion at Bungoma County Referral Hospital, Kenya

**DOI:** 10.11604/pamj.2021.38.280.22327

**Published:** 2021-03-17

**Authors:** Phidelis Maruti Marabi, Stanslaus Kiilu Musyoki, Angela Amayo

**Affiliations:** 1 Bungoma County Referral Hospital, Bungoma, Kenya,; 2 School of Health Sciences, Kisii University, Kisii, Kenya,; 3 Department of Human Pathology, University of Nairobi, Nairobi, Kenya

**Keywords:** Blood transfusion, cellular changes, storage, Kenya

## Abstract

**Introduction:**

during the storage of transfusion blood, it may undergo a series of cellular changes that in speculation could be the reason behind the risk of using prolonged stored blood. It's important therefore to monitor the cellular changes that may reduce its survival and function. The objective was to assess the cellular changes in whole blood stored for transfusion at Bungoma county referral hospital.

**Methods:**

a single center, prospective and observational study design involving 20 randomly selected donor blood units in citrate phosphate dextrose adenine (CPDA-1) anticoagulant was employed, cellular changes were evaluated for 35 days. The changes were tested using the Celtac F Haematology analyzer. Statistical Analysis of variance was employed in the descriptive statistics. All the investigation was executed using statistical package for social sciences (SPSS V.23). Results were regarded as significant at P<0.05. Results were presented in tables and charts.

**Results:**

at the end of the 35 days blood storage at blood bank conditions, WBC, RBC, platelets counts and MCHC decreased significantly (P<0.0001, =0.0182, <0.0001, =0.0035). The MCV, HCT and MCH increased significantly (P <0.0001, =0.0003, =0.0115) while HGB had insignificant variance (P =0.4185).

**Conclusion:**

platelets, WBC, RBC counts, and indices are significantly altered in stored blood especially when stored over two weeks based on most of the cellular components analyzed in this study. The study, therefore, recommends the utilization of fresh blood to avoid the adverse outcome of cellular changes of reserved blood.

## Introduction

Blood is a composite tissue constituting cell and non-cell elements that perform multiple roles [1]. The non-cell elements comprise the plasma and its derivatives. The cell components are made up of WBCs, PLTs, and RBCs [1]. Blood to be transfused is kept for up to 35 to 42 days at 2-6°C in preservatives such as citrate phosphate dextrose adenine (CPDA) [2].

During storage, blood experience a sequence of cellular changes which minimize their lifespan and purpose, and the most affected product is whole blood [3]. The deterioration in blood and cellular constituents happen almost immediately it is removed from the donor and recipients in need of transfusions rely on the blood and blood components safety and potency [4]. To minimize the dangers linked with blood transfusion, advanced anticoagulants, additive solutions, red blood cell membrane stabilizers, preservatives, and bags were manufactured [5]. Even with these developments, several changes in blood stored for transfusion have been encountered and referred to as ‘red blood cell storage lesions’.

After transfusion integral hemodynamic is similar for current and stored cells; nonetheless, micro-vascular hemodynamic are acutely affected by stored cells which minimizes blood movement and oxygen transport. Furthermore, the existence of stored cells in the bloodstream alter cell-cell and cell-wall interchanges and modify the cell [3]. The oxidative injury manifests red cells extra vulnerable to stress as indicated by increased osmotic fragility in the course of the storage and resultant discharge of haemoglobin (HGB) and intracellular enzymes such as lactate dehydrogenase (LDH) into the floating plasma [6].

Even though RBCs might be stored at 2-6°C for up to forty-two days before transfusion, less is understood of how changes to RBCs in the course of storage might alter their attachment properties [7]. Some studies have shown that stored RBCs show radical deformability changes in the course of blood storage at 2-6°C. Studies have denoted that the distortion index of RBCs does not vary substantially during blood storage at 2-6°C. Nonetheless, radical differences prevail in time constants and circularity distribution widths, which can be utilized to estimate stored red blood cell quality or age [8]. During blood storage at 2-6°C, glycolysis is retarded and, as acid pile up, the amount of ATP reduces and the structure of the RBC is bit-by-bit changed from disc-shaped to echinocytic shapes [9].

The release of unbound haemoglobin following red blood cells lysis during blood storage and its effect on the intravascular nitric oxide metabolism after transfusion has been considered a predominant role [10]. Studies have denoted that transfusion of long-stored blood is linked with a rise in plasma unbound haemoglobin and hunting of nitric oxide in vitro [11]. In line with this discovery, elevated unbound haemoglobin levels in patients with chronic and severe hemolysis have been connected with reduced nitric oxide bioavailability within the micro-capillary bed, reduced organ perfusion, and raised organ injury [12]. Similarly, transfusion of stored blood pints may increase unbound haemoglobin levels in recipients after transfusion -for example, as a result of a pre-mature intravascular burst of the transfused RBCs, or because of the transfusion of the unbound haemoglobin -containing storage medium [13]. Consistent with this hypothesis, studies have shown that transfusing of unbound haemoglobin containing stored blood cause a significant rise of blood pressure in rats that is correlated with the unbound haemoglobin levels in the stored blood [14].

With prolonged storage, there is a shortage of ATP, then the pumps may not be able to maintain the ionic homeostasis of the red blood cell, leading to changes in shape and mean cell volume (MCV), haematocrit (HCT), mean cell haemoglobin (MCH) and mean cell haemoglobin concentration (MCHC). The water influx to the cytosol gives rise to the swelling of erythrocytes during storage and the non-existence of selective channels of performance as done in the spleen are associated with the change in shape and volume of the red blood cell during storage [15].

Studies have revealed that white blood cells have a short life span in stored blood (only a few hours) and transfusion after 24 hours of storage has been proved to be ineffective in raising the WBC count of patients [1]. Many studies have revealed that during storage the total WBC count decreases, and they associate this count reduction to degeneration of the granulocytes [1]. Studies have denoted that WBC depletion in the course of blood storage has been connected with ATP depletion and white blood cells being used in the development of micro-aggregates, which are a mixture of white blood cells, platelets, fibrin, cold insoluble globulin, and cellular debris formed during storage [16].

Studies show that in the course of storage, changes take place in both platelet and storage device, which may lead to the activation of platelet and malfunction [17]. The stimulation of Platelets in the course of storage of blood leads to Platelet-white blood cell aggregates (PLAs) accumulation that introduces WBC apoptosis. Pro-coagulant action, likely correlated with micro-particles from apoptotic white blood cells, might lead to harmful properties of stored blood [18]. Results from some studies indicate that canine platelets survive when stored at room temperature for up to eight hours in CPDA-1 treated whole blood. However, a gap still exists as to whether there are changes after 8 hours of storage [19].

The establishment of blood storing arrangements allows time between collection of blood and transfusion to be increased. This time increase has allowed the decentralization of blood donation utilities with successive savings and advancements in the accessibility of blood components. However, the accessibility of blood storage increases the question of to what extend blood components can and should be stored and to what extent are they safe and potent [20].

Elongated blood storage increases mortality, serious infections, and multi-organ failure after transfusion; however, the causes of these remain unknown [21]. According to records in Bungoma County Referral Hospital, a monthly average of 2-3% of patients reacts to transfused blood especially aged blood (>20 days). Blood toxicity has been speculated to be a result of changes as blood ages which are not monitored during storage [22]. Despite this, little is understood about the changes that take place in the course of storage of blood cells during blood storage at Bungoma and at large. This study thus; determined the cellular, biochemical changes, and bacterial contamination in whole blood stored for transfusion so that transfusion safety is ensured.

This study assessed cellular changes in whole blood stored for transfusion at Bungoma County Referral Hospital in the western region of Kenya between February 2019 and August 2019.

## Methods

**Ethical considerations:** the study was cleared by the Ethical committee of Jaramogi Oginga Teaching and Referral Hospital (#ERC.IB/VOL.1/454) and authority to carry out the research was issued by the National Commission for Science, Technology and Innovation (#NACOSTI/P/19/32125/27143). Written informed consent was obtained from each donor after a brief explanation of what the study is about. Participation in the study was voluntary. Donor details were kept confidential by the exclusion of all forms of identification on the data collection tool and filled data tools were stored physically under lock and digitally with restricted password access on a computer. The donors were informed that their blood units if selected for the study, were not transfused to patients.

**Study area:** the study was carried out between February and August 2019 at Bungoma County Referral Hospital that is located in Bungoma County (coordinates 0.4213° N to 1.1477° N along the latitude and 34.3627° E to 35.0677° E along the longitude) in Western Kenya. This was a good study area because blood reactions among the patients who receive a blood transfusion were common. The hospital also has an accredited laboratory that is well-equipped with haematology equipment required for the study. The hospital has a blood donation centre that collects an averagely of 600 blood units monthly.

**Study design:** the study was a single center, prospective and observational and involved the collection of blood pints from normal volunteers and reserved under blood bank conditions then tested at day 0 which was regarded as baseline and then serially at day7, day14, day21, day 28, and day 35. The whole unit of blood was used in the study to ensure that the minimum storage condition for blood meant for transfusion was observed.

**Study population:** the sample size of the research was determined by Yamane Taro formulae for the finite population [23]. A sample size of 20 blood units was adopted for the study. A simple random sampling technique was used in this study where every 10^th^ sample was selected for the study to eliminate bias.

### Data collection

**Sample collection and analysis:** all blood units were collected according to blood transfusion donor guidelines as described by the World Health Organisation [24]. At baseline, samples were immediately separated from blood units collected from the volunteer donors to test for WBC count, RBC count, HGB level, MCV, HCT, MCH, MCHC, and Platelet count. Blood units were then stored at blood bank conditions of 2-6°C for 35 days with intermittent sampling at 7-day intervals to test for White blood cell count, Red blood cell count, and Haemoglobin level, Mean cell volume, Haematocrit, Mean Cell Haemoglobin, Mean Cell Haemoglobin Concentration and platelet count. The samples for testing were aseptically transferred from blood units to plain test tubes during separation, No, any other anticoagulant or additives were added to the separated samples. The separated samples were brought to the right temperature as per the manufacturer's instructions before analysis. The laboratory results were recorded on a standard data collection form developed for this study. Cellular changes (Including RBC count, WBC count, and platelet count, HCT, and MCV) and haemoglobin changes were tested using Celtac F MEK-8222 haematology analyzer (NIHON KOHDEN Corporation, 1-31-4 Nishiochiai, Shinjuku-ku, Tokyo 161-8560, Japan). Briefly, the procedure involved pressing numerical on the screen to enter sample identification details; placing well-mixed samples on the autoloader; pressing the start icon on the screen to start sample aspiration and analysis; the results were displayed on the screen and automatically printed immediately the instrument completed the analysis.

The normal ranges are White blood cells count: 3.5-10.0 x 10g/L; Red blood cells: 4.50-6.50 x 10g/L; Haemoglobin: 12.5-16.5g/dL; Haematocrit: 25.0-45.0%; Mean cell volume: 70.0-100.0 fL; Mean cell haemoglobin concentration: 31.0-38.0 g/dL; and platelets: 130-400 x10g/L.

**Quality Assurance of the data:** to ensure that quality of data collected, pre-donation requirements ( such as ensuring that only donors with a haemoglobin level of 12.5g/dL and above, weighing 50 kilograms and above, without a history of recent (12 months) transfusion, that have not recently donated blood (3 months after the last donation), without history and signs of malignancy, without signs and symptoms of sickle cell disease, without signs and symptoms of polycythaemia Rubra Vera and without a history of haemophilia and other coagulation disorders donate blood) were followed and only blood units that met these criteria were used. A qualified phlebotomist collected the blood samples, ensuring that the right quantity of between 450 to 500ml was collected. The pints were transported and stored as per blood transfusion guidelines at 2-6°C. Sample aliquots at the various study points were brought to the optimum temperature as per the manufacturer's instructions before testing. Samples were analyzed in duplicates for each sample and an average was computed to ensure accuracy. Both external and internal quality controls for haematology tests were verified and ensured during the study. Recorded results were verified by second personnel to ensure accuracy. The laboratory is enrolled in the Human Quality assessment services (HUQAS) External Quality Assurance Scheme for haematology scope including WBC count, RBC count, HGB level, MCV, HCT, MCH, MCHC, and Platelet count. The haematology scope is also accredited by the Kenya Accreditation Services (KENAS) which further assured the quality of data collected.

**Data management and analysis:** the data was stored in Microsoft Excel (Microsoft Corporation, Redmond, Washington, United States). The interrogation was done using the Statistical Package for the Social Sciences (SPSS V.23) (IBM Corporation, Chicago, Illinois, United States). Descriptive statistics (frequencies, mean, and standard deviation) were used to describe the data. The trends of the cellular changes were shown using line plots. Analysis of variance (ANOVA) was used to establish if there were significant cellular changes in transfusion blood at baseline and each study point compared to the baseline for 35 days of storage. Findings were considered significant at p<0.05. Tukey´s Honest Significant Difference test was used to collate all feasible pairs of means. Results were presented as tables and charts.

## Results

**Figure 1:**
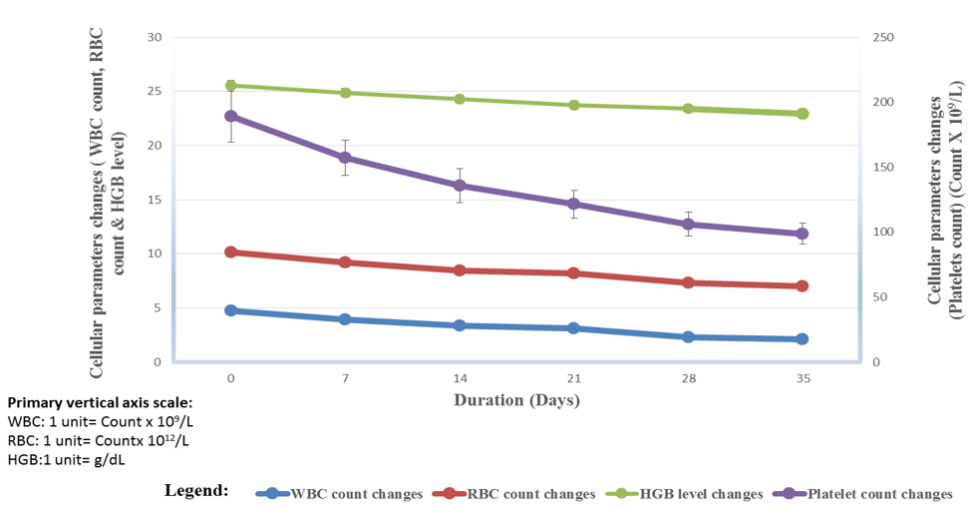
the trend of WBC, RBC, HGB and platelets changes in the progressing storage period of transfusion blood at Bungoma County Referral Hospital, Kenya. (Key: WBC=White Blood Cell (Count × 10^9^/L), RBC=Red Blood Cell (Count × 10^12^/L) and HGB= Haemoglobin (g/dL)

To show the trends of the cellular changes in progressing storage period of blood, the mean of white blood cell (WBC) counts, red blood cell (RBC) counts, haemoglobin (HGB) levels and platelet counts were measured at baseline (Day0), First week (Day7), second week (Day 14), third week (Day21), fourth week (Day 28) and firth week (Day 35) of storage for the samples and are illustrated in Figure 1. The white blood cell count demonstrated a decreasing trend from 5.85x10^9^/L±0.41 to 2.12±0.14x10^9^/L indicating that; white blood cells during the 35 days storage reduce; RBC count demonstrated a slightly decreasing trend from 5.36±0.41 x10^12^/L to 4.91±0.40 x10^12^/L indicating that red blood cells during the 35 days storage are slightly decrease; HGB level demonstrated a slightly increasing trend from 15.47±0.45 g/dL to 15.89±0.25 g/dL indicating that haemoglobin level during the 35 days storage is slightly raised; while platelet count demonstrated a decreasing trend from 188.90±19.67x10^9^/Lto93.80±8.26 x10^9^/L indicating that platelets during the 35 days storage are depleted (Figure 1).

**Figure 2:**
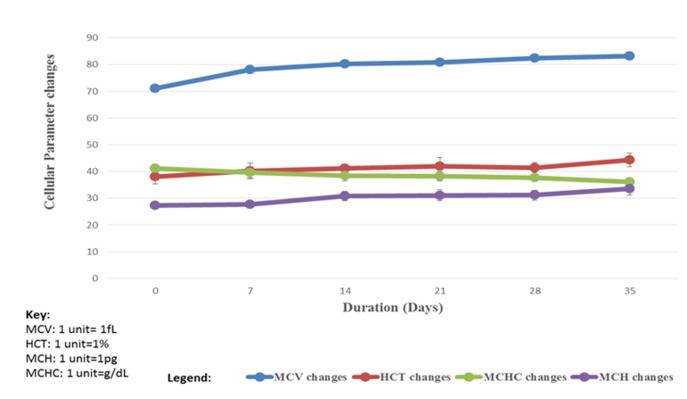
the trend of red blood cells indices (MCV, HCT, and MCH &MCHC) changes in the progressing storage period of transfusion blood at Bungoma County Referral Hospital, Kenya. (Key: MCV= Mean Cell Volume (fL), HCT= Haematocrit) (%), MCH=Mean Cell Haemoglobin (pg) and MCHC= Mean Cell Haemoglobin Concentration (g/dL)

Trends of Red blood indices were also evaluated and shown in Figure 2. The MCV demonstrated an increasing trend of 71.14±1.0fL to 83.2±1.25fL indicating that the mean cell volume during the 35 days storage is raised; HCT demonstrated a slightly increasing trend of 38.08±2.73% to 44.33±2.49% indicating that haematocrit during the 35 days storage is slightly raised; MCH demonstrated a slightly increasing trend of 27.42±0.71pg to 33.49±2.17pg indicating that mean cell haemoglobin during the 35 days storage is slightly raised; while MCHC demonstrated a slightly decreasing trend of 41.14g±1.27 /L to 36.08±1.01 g/L indicating that mean cell haemoglobin concentration is slightly reduced. (Figure 2).

This study observed that white blood cells count changes were non-significant after one week of storage, however significant reduction was observed after 14 days which further decreased significantly through to 35 days of storage. These results, therefore, indicate that; white blood cells during storage are significantly altered by the 7th day. Red blood cells count changes were insignificant up to three weeks (21 days) of storage, however significant reduction was observed at day 28 and further significantly decreased through to 35 days of storage. These results, therefore, indicate that; red blood cells during storage are significantly altered by the 28th day. Haemoglobin level estimation demonstrated an insignificant increase throughout the blood storage period. These findings, therefore indicate that haemoglobin level is non-significantly affected by storage. Mean cell volume demonstrated a significant increase from day 0 to day 35 storage period. These findings, therefore, indicate that; MCV is significantly increased during storage. Haematocrit demonstrated a slightly increase significant change being noted from day 14 of the storage period and continued throughout the remaining period of storage time. These findings, therefore, indicate that; haematocrit is significantly increased during storage. Mean cell haemoglobin demonstrated insignificant variance from day 0 to day 28; it, however, demonstrated significant variance (increase) at day 35 of storage time. These findings, therefore indicate that Mean cell haemoglobin is affected by storage from the 35th day of storage. Mean cell haemoglobin concentration demonstrated insignificant variance from day 0 to day 28; it, however, demonstrated significant variance (decrease) at day 35 of storage. These findings, therefore indicate that Mean cell haemoglobin concentration is affected by storage from the 35th day of storage. While platelets count demonstrated a significant decrease from day 14 and continued throughout the 35days storage period. These findings, therefore indicate that platelet count is significantly affected by storage from the 14th day of storage (Table 1).

**Table 1:** the cellular variance (change) throughout the blood storage period at Bungoma County Referral Hospital, Kenya, February to August 2019

		Day0 compared to the normal reference interval median	Day7 compared to baseline	Day14 compared to baseline	Day21 compared to baseline	Day28 compared to baseline	Day35 compared to baseline
**WBC**	Mean±SEM	5.85x10^9^/L±0.41	3.91x10^9^/L±0.27	3.35x10^9^/L±0.26	3.14x10^9^/L±0.23	2.28x10^9^/L±0.18	2.12x10^9^/L±0.14
	F	3.90	2.90	2.33	11.89	30.53	32.63
	**P-value**	**0.0777**	**0.965**	**0.0062**	**0.0014**	**<0.0001**	**<0.0001**
**RBC**	Mean±SEM	5.36x1012/L±0.41	5.26x1012/L±0.41	5.10x1012/L±0.40	5.03x1012/L±0.40	5.01x1012/L±0.40	4.91x1012/L±0.40
	F	0.20	0.21	1.85	3.20	3.77	6.09
	**P-value**	**0.3222**	**0.6473**	**0.81814**	**0.0816**	**0.0597**	**0.0182**
**HGB**	Mean±SEM	15.47g/dL±0.45	15.72g/dL±0.39	15.82g/dL±0.36	15.57g/dL±0.41	16.10g/dL±0.31	15.89g/dL±0.25
	F	1.10	0.18	0.37	1.32	1.32	0.67
	**P-value**	**0.6765**	**0.5456**	**0.2578**	**0.2578**	**0.2578**	**0.4185**
**MCV**	Mean±SEM	71.14±1.0fL	78.14±0.97fL	80.26±1.00fL	80.94±1.04fL	82.33±1.14fL	83.21±1.25fL
	F	0.83	17.12	31.31	37.39	50.15	52.53
	**P-value**	**0.3676**	**0.0002**	**<0.0001**	**<0.0001**	**<0.0001**	**<0.0001**
**HCT**	Mean±SEM	38.08±2.73%	40.21±2.94%	41.16±1.58%	40.92±3.37%	41.48±1.78%	44.33±4.49%
	F	1.89	1.86	4.00	3.36	5.15	16.30
	**P-value**	**0.1768**	**0.1810**	**0.0528**	**0.0707**	**0.0289**	**0.0003**
**MCH**	Mean±SEM	27.42±0.71pg	27.73±0.65pg	30.83±1.71pg	31.10±2.07pg	31.15±1.92pg	33.49±2.17pg
	F	8.49	3.36	3.36	2.82	3.30	7.05
	**P-value**	**0.0060**	**0.07544**	**0.0745**	**0.1013**	**0.0772**	**0.0115**
**MCHC**	Mean±SEM	41.14±1.27g/L	39.73±2.11g/L	38.50±2.00g/L	38.28±1.72g/L	37.74±1.38g/L	36.08±1.01g/L
	F	27.25	0.33	1.24	1.79	3.41	9.70
	**P-value**	**<0.0001**	**0.5716**	**0.2720**	**0.1885**	**0.0724**	**0.0035**
**PLT**	Mean±SEM	188.90±19.67x10^9^/L	157.25±13.78x10^9^/L	135.70±12.91x10^9^/L	121.40±10.60x10^9^/L	10.00±9.10x10^9^/L	93.80±8.26x10^9^/L
	F	14.97	1.74	5.11	9.12	14.63	19.87
	**P-value**	**0.0004**	**0.1955**	**0.0296**	**0.0045**	**0.0005**	**0.0001**


Baseline=Day0, week1=Day7, week2=Day14, week3=Day21, week4=Day28, week5=Day35. WBC=White Blood Cell (Countx10^9^/L), RBC=Red Blood Cell (Countx1012/L), HGB= Haemoglobin (g/dL), MVCV: Mean Corpuscular Volume, HCT: hematocrit, MCH: mean corpuscular hemoglobin, MCHC: mean corpuscular hemoglobin concentration, PLT: Platelets, SEM: Standard Error of the mean

## Discussion

The present study has shown that there are cellular changes during the storage period. The white blood cells count changes were non-significant after one week of storage. However significant reduction was observed after 14 days which further decreased significantly through to 35 days of storage. These results, therefore, indicate that; White blood cells during storage are significantly altered by the 7^th^ day. Factors that contribute to the white blood cells reduction during blood for transfusion storage could be loss viability because of ATP depletion. More so, leukocytes are used in the formation of white blood cell- platelet micro-aggregates, which are a mixture of white blood cells, platelets, fibrin, cold globulin, and cellular debris formed during storage (Ahmed, 2008). The clinical significance of this finding is that stored blood for transfusion could be particularly ineffective as a clinical tool in the management of aplastic anaemia and other leucopenic patients since the most critical establishment in these conditions is almost always neutropenia [25]. These findings do compare with findings of a study done in Braithwaite Memorial Specialist Hospital (BMSH), Port Harcourt, Rivers State, Nigeria which demonstrated that at 28 days, there were significant changes in white blood cell differential and absolute counts [26]. These results also concurred with the findings of another study carried out in Aminu Kano Teaching Hospital, Kano, Nigeria which illustrated that the percentage fall from day-zero to day-thirty five was 97% for white blood cells [27]. These findings also concurred with findings of another study done in the Veterinary Transfusion Research Laboratory, 85 University of Milan, Italy which showed that there was a statistically significant drop in WBC count after storage for 35 days of transfusion blood [28]. Another study done in L. N. Medical College and J. K. Hospital, Bhopal, India which demonstrated that WBC count constantly decreased throughout the 28 days storage period also concurred with these findings (Bhargava, Gupta, Vivek, & Khare, 2016). To the strength of the findings from the present study, white blood cells count monitoring during blood for transfusion storage intending to improve blood transfusion efficacy and safety and is recommended.

In the current study, the red blood cells count changes were insignificant up to three weeks (21 days) of storage. However significant reduction was observed at day 28 and further significantly decreased through to 35 days of storage. These results, therefore, indicate that; Red blood cells during storage are significantly altered by the 28^th^ day. The current findings can be explained by the fact that the systemic and biochemical changes that red blood cells go through in the course of storage are anticipated to be instrumental to the drop in red blood cell count as storage span increase [29]. During blood storage at 2-6°C, glycolysis is reduced and as acid level increase, the amount of ATP reduce, and the structure of the red cell is slowly changed from discoid to echinocytic shape [30]. If there is a shortage of ATP, then the pumps (co-transporters) may be unable to maintain the ionic homeostasis of the cell, leading to changes in red blood shape and volume [6]. The number of undamaged red blood cells that remain in a prolonged-stored blood unit before transfusion is not known and warrants additional research. A human red blood cell has a lifecycle of about one hundred and twenty days [31]. In normal conditions, about 2.4 million new red blood cells are generated per second with the concomitant eviction of an equivalent quantity of senescent red blood cells from the bloodstream. Hence, human blood constitutes reds blood cells that vary from zero to one hundred and twenty days of age, which is identical to a pint of freshly collected blood [32]. This experience may likely be suggestive of some level of cell selection where older and more labile cells die initially rapidly, thus leaving a cohort of younger and more stable cells that die later at a much slower rate [27]. The clinical significance of these findings is that adjustment in architecture from basic bio-concave rings to echinocytic red blood cells makes the cells easier to clump, increasing the possibility of blocking the microcirculation, leading to tissue ischemia (Adam, 2015). These less elastic red blood cells are not able to cross tiny micro-vessels of the micro-circulation, leading to reduced oxygen transport since the aerated red cells can´t cross the end organ capillary beds (Yalcin, 2014). Transfused stored RBCs can provoke a pro-inflammatory response by the cytokines and eicosanoids. Storage lesions also promote adhesion to endothelial cells, complement system activation, and changes in coagulability. These effects also damage the endothelial lining to cause capillary leakage [9]. The pro-inflammatory nature of stored RBCs has been correlated with an increased fatality, multiple organ failure, thrombosis, and protracted hospital stay [33].

These findings compare with findings from the previous study done in Port Harcourt, Rivers State, Nigeria which showed no statistically significant changes in red blood cell count in the course of the 28 days storage period [26]. These findings also compare with the findings of a study conducted in Bhopal, India which demonstrated no significant changes in red blood cell count during the 28 days storage period [16]. These findings also compare with the findings of a study conducted in Rohtak, India which demonstrated no significant changes in red blood cell count during the 28 days storage period [34]. However, the finding of this study contradicts the findings of a study done in L. N. Medical College and J. K. Hospital, Bhopal, India which showed that RBC count increased during the 28 day storage period (Bhargava *et al*., 2016). These findings also differ from the findings of a study done in São João Hospital, Porto, Portugal which showed that the RBC count kept unchanged throughout the 42 days of storage [5]. These findings also differ from the findings of a study done in Sanjay Gandhi Memorial Hospital, Rewa, India which demonstrated that red blood cell count showed no significant change during the 35 days storage period [25]. In light of the findings from the present study, red blood cells count monitoring during blood for transfusion storage intending to improve blood transfusion efficacy and safety is recommended.

In the current study, haemoglobin level estimation demonstrates an insignificant increase throughout the blood storage period. The slight increase in Haemoglogin level can be explained by the fact that during storage, the byproducts of glycolytic metabolism, lactic acid, and proteins accrue, which in vivo are readily removed from the bloodstream, remain and give rise to physical changes and cell lysis releasing unbound haemoglobin into plasma [35]. The clinical significance of these findings is that transfusing older pints with unbound haemoglobin has transfusion-related harm especially for patients who have a history of unbound haemoglobin in their circulation [10]. Unbound haemoglobin may trigger vasoconstrictive, pro-oxidative, and pro-inflammatory events that have transfusion-related harm to the transfused patient [9]. Our findings do compare with a previous study in São João Hospital, Porto, Portugal which showed that the haemoglobin amount remained un-varied in the course of the 42 days of reservation [5]. However, our findings contrast a previous study done in the Department of Pathology, S.S. Medical College Rewa, and India which demonstrated that haemoglobin concentration gradually decreased during the 35 day storage period [25]. In light of the findings from the present study, haemoglobin monitoring during blood for transfusion storage and conditions of patients that might lead to the release of unbound haemoglobin in their circulation need be considered before indicating a transfusion to improve blood transfusion efficacy and safety and is recommended.

In the present study, HCT demonstrates a slightly increasing trend with the significant change being noted from day 14 of the storage period and continues throughout the remaining period of storage time. The current findings can be explained by the fact that the increase in haematocrit reflects the morphological alterations that take place during blood storage (Bosman *et al*., 2008). The clinical significance of these findings is that increased morphological alteration minimizes the potency of transfused blood by increasing the speed of elimination of transfused cells by the macrophage [2]. These results compare with previously documented findings São João Hospital, Porto, Portugal which demonstrated that HCT increased from day 0 to day 14 and remained stable afterward [5]. Our findings also compare with findings from another study done in Doha, Qatar that demonstrated a significant HCT increase after 35 days of blood storage [30]. However, the current study contrasts the findings of a study done in Obafemi Awolowo University Teaching Hospital Complex, Ile-Ife, Osun State, Nigeria which showed a significant fall of haematocrit [36]. Our findings also contrast findings of a study done conducted in Braithwaite Memorial Specialist Hospital (BMSH), city of Port Harcourt, Rivers State, Nigeria which demonstrated an insignificant change of HCT throughout the 28 days storage period [26]. To the strength of the findings from the present study, HCT monitoring during blood for transfusion storage and use of blood less than 14 days to improve blood transfusion efficacy is advocated for.

In the current study, MCH and MCHC demonstrate insignificant variance from day 0 to day 28; it, however, demonstrates significant variance (increase and decrease respectively) at day 35 of storage time. The changes in MCH and MCHC appear to be the result of a deregulated mechanism of cell volume, which expounds the increase in the volume of the RBCs, the increasing hypocromia, and the anisocytosis. Hence, the decrease in MCHC does not result from a reduction in hemoglobin concentration but an increase in cell volume [5]. These results compare with those findings documented in a study done in Braithwaite Memorial Specialist Hospital (BMSH), city of Port Harcourt, Rivers State, Nigeria which showed that the MCH and MCHC changes were insignificant during the 28 days of storage [26]. However, the results contrast the findings of a study done in Iran which demonstrated that MCH decreased during the storage period [37]. In light of the findings from the present study, MCH and MCHC monitoring during blood for transfusion storage and use of fresh blood less than 28 days with a focus on improving blood transfusion efficacy is recommended.

In the present study, Platelets demonstrates a significant decrease from day 14 and continues throughout the 35days storage period. The current findings can be explained by the fact that the cell loose viability owing to ATP depletion in addition to platelet utilization due to micro-aggregates development [27]. The clinical significance of these findings is that this may expose patients to possible decreases in platelets effectiveness as well as likely increases in adverse incidences in addition to transfusion-related sepsis, such as inflammation and/or immune-mediated incidences. Seriously sick patients, including post-cardiac surgery patients and haematology/oncology patients, may be specifically vulnerable to platelets' adverse incidences because of their pre-transfusion inflammatory state [17]. These results are in comparison with those obtained in a study done in Aminu Kano Teaching Hospital, Kano, Nigeria which demonstrated a constant decrease of platelets throughout the 35 days storage period (Ahmed, 2008). Our findings also compare with those results abstained in a study done in L. N. Medical College and J. K. Hospital, Bhopal, India which demonstrated that platelet decreased significantly during the storage period [16]. Our findings also compare with the findings documented in a study done in Nigeria which demonstrated 86.2% platelet count fall from day 0 to day 28 storage times [27]. Our findings, however, contrast the findings of a study done in Nigeria which demonstrated insignificant platelet count variance throughout the 28 days storage period [26]. In light of the findings from the present study, platelet count monitoring during blood for transfusion storage to improve blood transfusion efficacy and safety is recommended. Overall, together with other parameters, cellular changes in stored blood for transfusion should be keenly monitored putting into consideration the patient to be transfused, and the clinical indication of the blood.

**Limitations:** the current study assessed cellular changes in blood for transfusion stored at 2-6°C at a single facility, which may not be representative of the whole country.

## Conclusion

Platelets, WBC, RBC counts, and indices are significantly altered in stored blood especially when stored over two weeks based on most of the cellular components analyzed in this study. The clinical consequence of this is that long-reserved whole blood would be distinctly worthless as a clinical tool in the management of blood disorders. Fresh whole blood of not more than two weeks is recommended as a better choice of transfusion of whole blood however blood components may vary depending on the level of changes in each blood parameter or indices.

### What is known about this topic

- Evidence that patients react adversely following transfusion is available;
- Evidence that red blood cells experience harmful changes during storage is available;
- Evidence that transfusing older, stored blood might lead to increased fatality; severe infections, multiple organ breakdown, thrombosis, and lengthened hospital stay is available.

### What this study adds

- The study has identified what storage extend is stored blood for transfusion potent and safe;
- The study has shed light on the need to carefully monitor the cellular changes during blood storage;
- The study has shed light on the need to transfuse patients who have a history of unbound haemoglobin in their circulation with caution.

## References

[1] Nuaimy K (2008). Haematological Changes in Stored Blood. J Educ Sci.

[2] Oyet C, Okongo B, Onyuthi RA, Muwanguzi E (2018). Biochemical changes in stored donor units: implications on the efficacy of blood transfusion. J Blood Med.

[3] Yalcin O, Ortiz D, Tsai AG, Johnson PC, Cabrales P (2014). Microhemodynamic aberrations created by transfusion of stored blood. Transfusion.

[4] Isbister J (2003). “Is the Clinical Significance of Blood Storage Lesions Underestimated?. Transfus Altern Transfus Med.

[5] Diana Noguira EC, Susana Rocha, Estela Abreu (2015). Biochemical and Cellular Changes in Leukocyte-Depleted Red Blood Cells Stored for TransfusionNo Title. Transfus Med Hemotherapy.

[6] Orlov K (2015). The pathophysiology and consequences of red blood cell storage. Anaesthesia.

[7] Anniss AM, Sparrow RL (2006). Storage duration and white blood cell content of red blood cell (RBC) products increases adhesion of stored RBCs to endothelium under flow conditions. Transfusion.

[8] Zheng Y, Chen J, Cui T, Shehata N, Wang C, Sun Y (2012). Characterization of red blood cell deformability change during blood storage. Lab Chip.

[9] Yoshida PD (2019). Red blood cell storage lesion: causes and potential clinical consequences. Blood Transfus.

[10] Iris C, Vermeulen Windsant, Norbert C J de Wit, Jonas T C Sertorio, Erik A M Beckers, Jose E Tanus-Santos, Michael J Jacobs, Wim A Buurman (2012). “Blood transfusions increase circulating plasma free hemoglobin levels and plasma nitric oxide consumption: a prospective observational pilot study”. Crit Care.

[11] Finney SJ (2012). “Free haemoglobin in “old” transfused blood-baddy or bystander?”. Crit Care.

[12] Reiter G, Wang Tanus-Santos, Hogg Cannon Schechter (2020). “Cell-free hemoglobin limits nitric oxide bioavailability in sickle-cell disease”. Nat Med.

[13] Gladwin K-S (2009). “Storage lesion in banked blood due to hemolysis-dependent disruption of nitric oxide homeostasis”. Curr Opin Hematol.

[14] Chenell Donadee, Nicolaas J H, Raat Tamir Kanias, Jesús Tejero, Janet S Lee, Eric E Kelley (2011). Nitric oxide scavenging by red blood cell microparticles and cell-free hemoglobin as a mechanism for the red cell storage lesion. Circulation.

[15] Bosman W, Lasonder Groenen-Döpp, Willekens (2012). The proteome of erythrocyte-derived microparticles from plasma: new clues for erythrocyte aging and vesiculation. J proteomic.

[16] Bhargava P, Gupta R, Vivek Khare (2016). CPDA-1 Stored Blood Induced Effect on Hematological and Biochemical Parameter up to 28 DaysNo Title. Adv path lab Med.

[17] Cécile Aubron, Andrew WJ Flint, Yves Ozier, Zoe McQuilten (2018). Platelet storage duration and its clinical and transfusion outcomes: a systematic review. Crit Care.

[18] Keating FK, Butenas S, Fung MK, Schneider DJ (2011). Platelet-white blood cell (WBC) interaction, WBC apoptosis, and procoagulant activity in stored red blood cells. Transfusion.

[19] Crowther M, Ford I, Jeffrey RR, Urbaniak SJ, Greaves M (2000). Quality of harvested autologous platelets compared with stored donor platelets for use after cardiopulmonary bypass procedures. Br J Haematol.

[20] Zimrin AB, Hess JR (2009). Current issues relating to the transfusion of stored red blood cells. Vox Sanguinis.

[21] Chelsea Sheppard CH, Cassandra Josephson (2005). Bacterial Contamination of Platelets for Transfusion: Recent Advances and Issues. Labmedicine.

[22] Patrick Burger, Elena Kostova, Esther Bloem, Petra Hilarius-Stokman, Alexander B Meijer, Timo K van den Berg (2013). Potassium leakage primes stored erythrocytes for phosphatidylserine exposure and shedding of pro-coagulant vesicles. Br J Haematol.

[23] Israel GD (1992). Determining Sample Size. Florida Coop Extention Serv, Fact Sheet PEOD-6.

[24] WHO (2012). Blood donor selection: guidelines on assessing donor suitability for blood donation. Blood Donor Sel.

[25] Batham N (2018). Evaluation of haematological parameter in stored CPDA-1 whole blood. Int J Appl Res.

[26] Teddy C, Adias Beatrice, Moore-Igwe Zaccheaus A, Jeremiah (2012). “Storage Related Haematological and Biochemical Changes of CPDA-1 Whole Blood in a Resource-Limited Setting. J Blood Disord Transfus.

[27] Ahmed O (2008). Cellular Changes in Stored Whole Blood and the Implication on Efficacy of Transfusion Therapy in Nigeria. Internet J. Third World Med.

[28] Eva Spada DP, Roberta Perego, Luciana Baggiani (2018). No Haematological and morphological evaluation of feline whole blood units collected for transfusion purposes Title. J Feline Med. Surgery.

[29] (2017). María García-Roa María Del Carmen Vicente-Ayuso Alejandro M Bobes Alexandra C Pedraza Ataúlfo González-Fernández María Paz Martín, Red blood cell storage time and transfusion: current practice, concerns and future perspectives. Blood Transfus.

[30] H Mustafa, Marwani Mamdouh Nasr, Abdulla Kano (2016). “Time-Dependent Assessment of Morphological Changes: Leukodepleted Packed Red Blood Cells Stored in SAGM”. Biomed Res Int.

[31] (2017). Clemente Fernandez Arias, Cristina Fernandez Arias, How do red blood cells know when to die?. R Soc Open Sci.

[32] Wei-Wei Tuo W-JL, Di Wang (2014). How Cell Number and Cellular Properties of Blood-Banked Red Blood Cells of Different Cell Ages Decline during Storage. PLoS One.

[33] Hod EA, Spitalnik SL (2011). Harmful effects of transfusion of older stored red blood cells: Iron and inflammation. Transfusion.

[34] Sonia Chhabra mg, Saurav Chaudhary, Sehgal Sunita Singh, Sen R (2017). “Changes in RBC and Platelet indices in CPDA stored BLOOD”. Int J Heal care Biomed Res.

[35] Arif M, Yadav Rehman (2017). Study of Hemolysis During Storage of Blood in the Blood Bank of a Tertiary Health Care Centre. Indian J. Hematol. blood Transfus.

[36] Oluyombo A, Uchegbwu Adegbamigbe (2013). Quantitative assessment of erythrocytes and leucocytes in CPD-A stored blood. Biomed Res.

[37] Ghezelbash G, Azarkeivan Pourfathollah, Deyhim Hajati (2018). Comparative Evaluation of Biochemical and Hematological Parameters of Pre-Storage Leukoreduction during RBC Storage. Hematol Oncol Stem Cell Res.

